# Expansion Microscopy on *Saccharomyces cerevisiae*

**DOI:** 10.17912/micropub.biology.000566

**Published:** 2022-05-04

**Authors:** Artemis G. Korovesi, Leonor Morgado, Marco Fumasoni, Ricardo Henriques, Hannah S. Heil, Mario Del Rosario

**Affiliations:** 1 Instituto Gulbenkian de Ciência, Oeiras, Portugal; 2 MRC-Laboratory for Molecular Cell Biology. University College London, London, United Kingdom

## Abstract

The unicellular eukaryote *Saccharomyces cerevisiae* is an invaluable resource for the study of basic eukaryotic cellular and molecular processes. However, its small size compared to other eukaryotic organisms the study of subcellular structures is challenging. Expansion microscopy (ExM) holds great potential to study the intracellular architecture of yeast, especially when paired with pan-labelling techniques visualising the full protein content inside cells. ExM allows to increase imaging resolution by physically enlarging a fixed sample that is embedded and cross-linked to a swellable gel followed by isotropic expansion in water. The cell wall present in fungi – including yeast – and Gram-positive bacteria is a resilient structure that resists denaturation and conventional digestion processes usually used in ExM protocols, resulting in uneven expansion. Thus, the digestion of the cell wall while maintaining the structure of the resulting protoplasts is a crucial step to ensure isotropic expansion. For this reason, specific experimental strategies are needed, and only a few protocols are currently available. We have developed a modified ExM protocol for *S. cerevisiae* , with 4x expansion factor, which allows the visualisation of the ultrastructure of the cells. Here, we describe the experimental procedure in detail, focusing on the most critical steps required to achieve isotropic expansion for ExM of *S. cerevisiae* .

**Figure 1. Yeast expansion microscopy overview:**
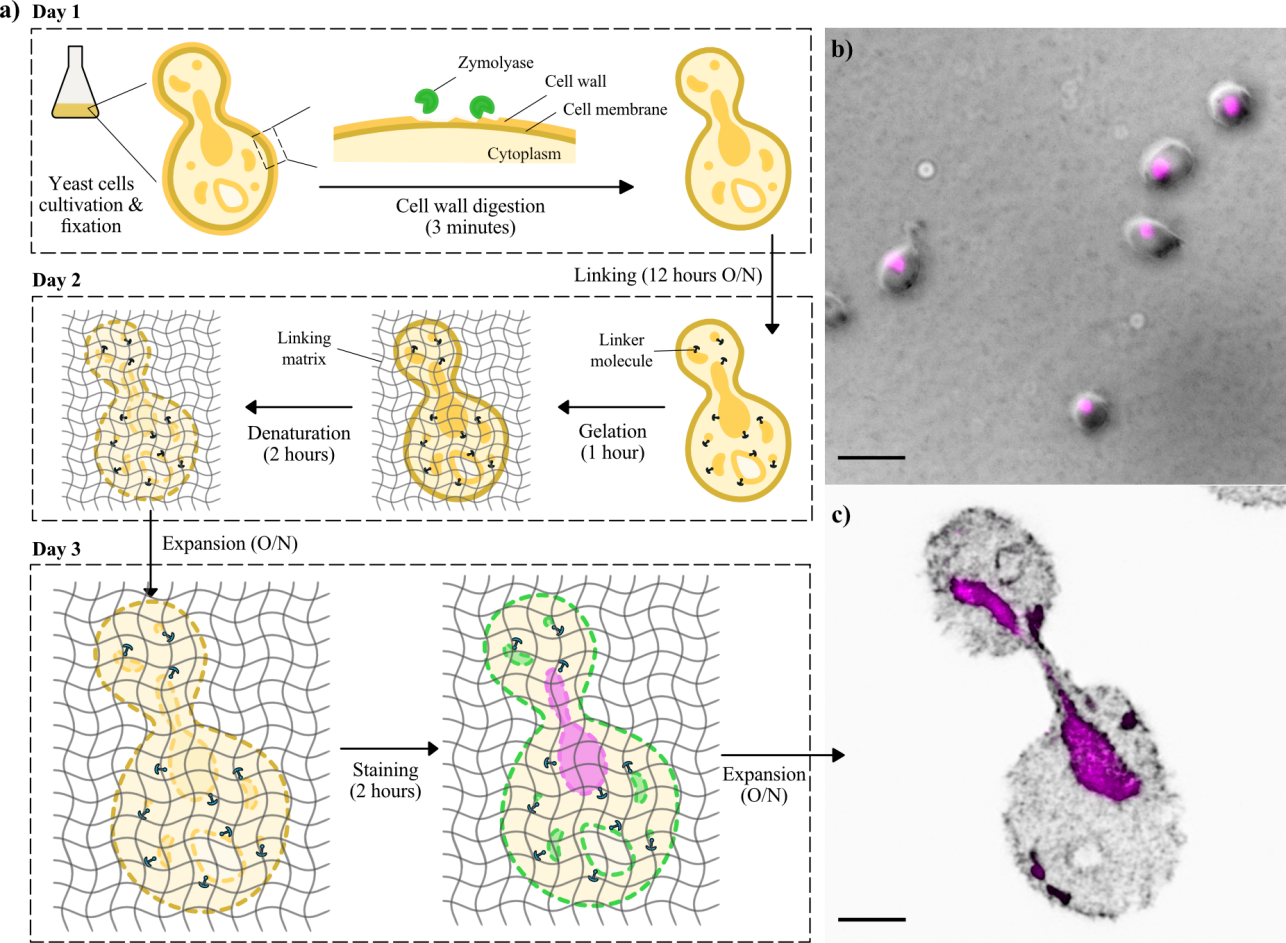
***a)*** *Scheme of expansion microscopy protocol workflow.* ***b)*** *Maximum intensity projection of DIC/DAPI composite image of yeast, acquired with a Nikon HCS using a 100x/1.45 oil objective.* ***c)*** *Maximum intensity projection of fluorescence image of post-expanded yeast, acquired with a Zeiss LSM 980 using a 40x/1.1 water objective and airyscan detection. Signals of DAPI and DIC/NHS-Ester are represented in magenta and grey, respectively. Scale bars: 5 µm. Samples were assessed by manual segmentation and measurement of the area and circularity between expanded (n=13) and non-expanded (n=27) specimens. The average expansion factor across all experiments was 3.52 ± 0.42 (mean ± standard deviation) while the circularity was retained within the level 20% of the population’s standard deviation.*

## Description

The unicellular eukaryote *S. cerevisiae* represents an invaluable resource for the study of basic eukaryotic cellular and molecular processes. The combination of a high genetic amenability, numerous genetic tools, and vast genomic resources makes it one of the most versatile model organisms, employed in a wide range of basic research disciplines. However, its small size compared to other eukaryotic organisms has limited its use for the study of sub-cellular structures. The diameter of unbudded yeast cells ranges approximately between 4 μm in haploids and 6 μm in diploids (Milo and Phillips 2015), complicating conventional diffraction-limited light microscopy approaches.

The last decade witnessed the rise of super-resolution (SR) techniques that enable sub-diffraction resolution fluorescence imaging of cellular structures. Expansion microscopy (ExM), for instance, allows for increasing imaging resolution by physically enlarging a fixed sample that is embedded and cross-linked to a swellable gel and is then expanded isotropically in water (Wassie *et al.* 2019). By doing so, fluorescently-labelled structures that previously would be too small or too close together to be distinguished can be resolved in 3 dimensions. The increased z-resolution can be exploited by z-sectioning, using confocal or light-sheet microscopy. In addition, ExM can also be used in combination with other super-resolution methods, such as airyscan confocal microscopy and Structured Illumination Microscopy (SIM), further enhancing resolution. ExM holds great potential to study the cellular structures of yeast, especially when paired with pan-labelling techniques to visualise total protein content inside cells. ExM protocols present a robust tool to study cellular organelles, obtaining super-resolution results with the advantage of employing inexpensive and straightforward equipment compared to other techniques that require more complex and expensive setups, such as optical SR approaches and electron microscopy.

ExM can be applied in various organisms and cellular models, offering outstanding outcomes with expansion factors ranging from 4x to 20x (Faulkner *et al.* 2020, Truckenbrodt *et al.* 2018). However, certain cellular organelles are resistant to these processes, such as the cell wall of fungi and Gram-positive bacteria. These organisms present a cell wall composed of peptidoglycans or glycoproteins that are highly resistant to denaturation and conventional digestion processes. The digestion of the cell wall and the maintenance of the structure of the resulting protoplasts are crucial steps to achieve isotropic expansion. For this reason, specific experimental strategies are needed, and only a few protocols are currently available (Götz *et al.* 2020). In particular, only one study describes an ExM protocol applied to *S. cerevisiae* , showing different yeast structures in high resolution (Chen *et al.* 2021).

Here we describe an adapted and extended ExM protocol for *S. cerevisiae* (Figure 1) with a final expansion factor of ~3.5x. The major difference regarding to the protocol of Chen *et al.* , is the staining strategy. While the previously published protocol is based on immunolabeling to visualise and super-resolve specific target proteins, here, samples are stained with DAPI and the pan-labelling reagent NHS-Ester BODIPY FL. This allows to provide an overview of the ultrastructural context of the whole cells. However, both protocols complement each other and could be combined, in principle. Here, we have compiled detailed step-by-step instructions for ExM to visualise the ultrastructure of *S. cerevisiae* , supported by supplementary video documentation (Supplementary Video 1).

Briefly, the first day begins with the preparation and fixation of yeast cells, which includes the digestion of the cell wall with zymolyase. The generated protoplasts are mounted on the coverslip, and linking takes place overnight, during which linker molecules will attach to the sample’s proteins, acting as anchors for the next step. On the second day, the first step is gelation, where the sample is embedded in a monomer solution that polymerases and forms a matrix to which the anchor molecules will be connected. This is followed by the denaturation step, where the sample structures are disintegrated. The samples are then left expanding overnight in water, swelling the polymerised gel, which will push the anchored molecules apart in an isotropic manner. Finally, on the third day, staining with DAPI and NHS-Ester takes place. Samples are left again expanding overnight, and they can be imaged from the next day onwards.

Assessing the expansion factor and specimen cellular integrity following sample preparation is a critical step that requires consideration. During sample expansion, the denaturation/digestion steps facilitate isotropic expansion of the sample but different organelle structures might possess specific requirements that need to be identified during the experimental planning phase of the experiment. The simplest solution to assess specimen integrity and expansion factor is to acquire microscopy data of the same field of view and specimen before and after expansion. This process will allow the user to detect immediate changes in the sample but carries the complication of being difficult to execute since finding the same sample, particularly in yeast, can be difficult and time-consuming. Other options, such as the one included in this protocol, image multiple expanded and non-expanded cells and compare their features (area and circularity) as means to assess expansion (average circularity 0.892 ± 0.063 on expanded samples (mean ± standard deviation, n=13) and 0.906 ± 0.035 on non-expanded ones (n=27)). Individual organelle expansion should be obtained via specific labelling of the structure and determining before and after expansion sizes and compare it to the expansion factor of the whole cell.

## Methods

Preparation of stock solutions:

**Sorbitol buffer:**

● 1.2 M sorbitol solution in 0.1 M KH _2_ PO _4_ .

**Linking solution:**

● 0.1 mg/mL acryloyl X-SE in PBS. Store in aliquots at -20 °C.

**Monomer solution:**

Stocks:

● 38% sodium acrylate (w/w, diluted with ddH _2_ O): 25 g in 65.79 mL, store at -20 °C.

● 40% acrylamide stock: 20 g in 50 mL ddH _2_ O, store at -20°C.

● 2% N,N’-Methylenebisacrylamide : 0.2 g in 10 mL ddH _2_ O, store at -20 °C.

**Table:** 

Reagent	Final concentration
PBS	1x
Sodium acrylate*	19 g/100 mL
Acrylamide	10 g/100 mL
N,N’-Methylenebisacrylamide	0.1 g/100 mL

Store in 493 µL aliquots at -20 °C.

*Sodium acrylate is provided with variable quality levels. One should test it before using it: the reagent should be fully dissolved and not show impurities in the solution, in the form of precipitates or discolouring.

**Denaturation buffer:**

**Table:** 

Reagent	Final concentration
ddH2O	-
SDS	200 mM
NaCl	200 mM
Tris*	50 mM

Store in falcon tubes at -20 °C.

*Adjust pH to 9.

**YPD Media:**

1. Dissolve 5 g of yeast extract and 10 g of peptone in 375 mL of ddH _2_ O by manually stirring the flask and autoclave the solution.

2. Add 100 mL of ddH _2_ O, 20 mL of autoclaved 50% Glucose (Dextrose) and 5 mL 1% Adenine + 1% Tryptophan.

Coverslip cleaning

Notes: i) This procedure should be performed under a fume hood, and one should use safety gloves when handling chloroform. ii) The chloroform and NaOH solutions can be reused. iii) The preparation of NaOH is an exothermic reaction, thus the NaOH stock solution should be prepared on an ice bath. iv) Other coverslip cleaning protocols are available, such as a chloroform-free protocol (Pereira *et al.* 2015).

Coverslip poly-L-lysine coating

**Day 1**

Yeast cells cultivation and fixation

The *Saccharomyces cerevisiae* BY4741 strain ( *MATa his3Δ1 leu2Δ0 met15Δ0 ura3Δ0* ) was used in this protocol.

Cell wall digestion

* The length of the reaction provided is optimised for a population of exponentially growing cells. Samples collected in other conditions may require an adjusted treatment to optimise the cell wall digestion.

Mounting and fixating the cells on the coverslip

Linking

**Day 2**

Gelation

Note: Proceed quickly through steps 3 to 5, as after the gelation solution is prepared, it will polymerise in minutes. For this reason, it is recommended to proceed with these steps with a maximum of four samples each time. Reagents and slides are maintained on ice to prevent premature polymerisation.

**Table:** 

Volume	Reagents	Stock concentration	Final concentration
493 µL	Monomer solution	-	-
5 µL	APS	50%	0.5%
2.5 µL	TEMED	99%	0.5%
500 µL	Final Volume	-	-

Denaturation

Expansion

**Day 3**

NHS and DAPI staining

**Imaging day**

Immobilisation of the gel

## Reagents

**Table:** 

**Resources**	**Supplier**	**Article number**
Acrylamide	Sigma	A9099
Acryloyl-X SE	Invitrogen	A20770
Adenine	Sigma	A9126
APS - Ammonium Persulfate	Roth	9592.3
DAPI	Invitrogen	D1306
DMAA - N,N’-Methylenebisacrylamide	Sigma	M7279-25G
GA - Glutaraldehyde	Sigma	G5882-10X1ML
Glucose (Dextrose)	Sigma	G7021
KH2PO4 - Potassium dihydrogen phosphate	Sangon Biotech	A100781
NaCl	Roth	HN00.2
NHS-Ester	Thermo Fisher Scientific	D2184
Peptone	Thermo Fisher Scientific	211820
PFA – Paraformaldehyde	Thermo Fisher Scientific	43368
Poly-L-Lysine	Sigma	P8920
SDS – Sodium Dodecyl Sulfate	Sigma	L3771
Sodium acrylate	Sigma	408220
Sorbitol solution	Sangon Biotech	A100691
TEMED – N,N,N′,N′-Tetramethylethylenediamine	Sigma	T9281
Tris	Carl Roth	5429.1
Tryptophan	Sigma	T0254
Tween-20	Roth	9127.1
Yeast extract	Thermo Fisher Scientific	288620
Zymolyase enzyme	Zymo research	E1004
Attofluor™ Cell Chamber	Thermo Fisher Scientific	A7816
Coverslip (24 mm round #1.5)	Marienfeld	117640
Coverslip rack	Diversified Biotech	WSDR-1000
Razor blade	Carl Roth	CK08.1

## References

[R1] Chen L, Yao L, Zhang L, Fei Yn, Mi L, Ma J. 2021. Applications of Super Resolution Expansion Microscopy in Yeast. Frontiers in Physics. 9:650353. 10.3389/fchem.2021.640519 PMC8119759 33996746

[R2] Faulkner EL, Thomas SG, Neely RK (2020). An introduction to the methodology of expansion microscopy.. Int J Biochem Cell Biol.

[R3] Götz R, Panzer S, Trinks N, Eilts J, Wagener J, Turrà D, Di Pietro A, Sauer M, Terpitz U (2020). Expansion Microscopy for Cell Biology Analysis in Fungi.. Front Microbiol.

[R4] Milo, R., & Phillips, R. 2015. Cell Biology by the numbers (1st ed.). Garland Science.

[R5] Pereira PM, Almada P, Henriques R (2015). High-content 3D multicolor super-resolution localization microscopy.. Methods Cell Biol.

[R6] Truckenbrodt S, Maidorn M, Crzan D, Wildhagen H, Kabatas S, Rizzoli SO (2018). X10 expansion microscopy enables 25-nm resolution on conventional microscopes.. EMBO Rep.

[R7] Wassie AT, Zhao Y, Boyden ES (2018). Expansion microscopy: principles and uses in biological research.. Nat Methods.

